# Academic dishonesty when doing homework: How digital technologies are put to bad use in secondary schools

**DOI:** 10.1007/s10639-022-11225-y

**Published:** 2022-07-23

**Authors:** Juliette C. Désiron, Dominik Petko

**Affiliations:** Institute of Education, University of Zürich, Zürich, Switzerland

**Keywords:** Academic dishonesty, Digitally-supported cheating, Homework, Plagiarism, Secondary education

## Abstract

The growth in digital technologies in recent decades has offered many opportunities to support students’ learning and homework completion. However, it has also contributed to expanding the field of possibilities concerning homework avoidance. Although studies have investigated the factors of academic dishonesty, the focus has often been on college students and formal assessments. The present study aimed to determine what predicts homework avoidance using digital resources and whether engaging in these practices is another predictor of test performance. To address these questions, we analyzed data from the Program for International Student Assessment 2018 survey, which contained additional questionnaires addressing this issue, for the Swiss students. The results showed that about half of the students engaged in one kind or another of digitally-supported practices for homework avoidance at least once or twice a week. Students who were more likely to use digital resources to engage in dishonest practices were males who did not put much effort into their homework and were enrolled in non-higher education-oriented school programs. Further, we found that digitally-supported homework avoidance was a significant negative predictor of test performance when considering information and communication technology predictors. Thus, the present study not only expands the knowledge regarding the predictors of academic dishonesty with digital resources, but also confirms the negative impact of such practices on learning.

## Introduction

Academic dishonesty is a widespread and perpetual issue for teachers made even more easier to perpetrate with the rise of digital technologies (Blau & Eshet-Alkalai, 2017; Ma et al., 2008). Definitions vary but overall an academically dishonest practices correspond to learners engaging in unauthorized practice such as cheating and plagiarism. Differences in engaging in those two types of practices mainly resides in students’ perception that plagiarism is worse than cheating (Evering & Moorman, 2012; McCabe, 2005). Plagiarism is usually defined as the unethical act of copying part or all of someone else’s work, with or without editing it, while cheating is more about sharing practices (Krou et al., 2021). As a result, most students do report cheating in an exam or for homework (Ma et al., 2008). To note, other research follow a different distinction for those practices and consider that plagiarism is a specific – and common – type of cheating (Waltzer & Dahl, 2022). Digital technologies have contributed to opening possibilities of homework avoidance and technology-related distraction (Ma et al., 2008; Xu, 2015).

The question of whether the use of digital resources hinders or enhances homework has often been investigated in large-scale studies, such as the Program for International Student Assessment (PISA), the Trends in International Mathematics and Science Study (TIMSS), and the Progress in International Reading Literacy Study (PIRLS). While most of the early large-scale studies showed positive overall correlations between the use of digital technologies for learning at home and test scores in language, mathematics, and science (e.g., OECD, 2015; Petko et al., 2017; Skryabin et al., 2015), there have been more recent studies reporting negative associations as well (Agasisti et al., 2020; Odell et al., 2020). One reason for these inconclusive findings is certainly the complex interplay of related factors, which include diverse ways of measuring homework, gender, socioeconomic status, personality traits, learning goals, academic abilities, learning strategies, motivation, and effort, as well as support from teachers and parents. Despite this complexity, it needs to be acknowledged that doing homework digitally does not automatically lead to productive learning activities, and it might even be associated with counter-productive practices such as digital distraction or academic dishonesty. Digitally enhanced academic dishonesty has mostly been investigated regarding formal assessment-related examinations (Evering & Moorman, 2012; Ma et al., 2008); however, it might be equally important to investigate its effects regarding learning-related assignments such as homework. Although a large body of research exists on digital academic dishonesty regarding assignments in higher education, relatively few studies have investigated this topic on K12 homework. To investigate this issue, we integrated questionnaire items on homework engagement and digital homework avoidance in a national add-on to PISA 2018 in Switzerland. Data from the Swiss sample can serve as a case study for further research with a wider cultural background. This study provides an overview of the descriptive results and tries to identify predictors of the use of digital technology for academic dishonesty when completing homework.

### Prevalence and factors of digital academic dishonesty in schools

According to Pavela’s (1997) framework, four different types of academic dishonesty can be distinguished: cheating by using unauthorized materials, plagiarism by copying the work of others, fabrication of invented evidence, and facilitation by helping others in their attempts at academic dishonesty. Academic dishonesty can happen in assessment situations, as well as in learning situations. In formal assessments, academic dishonesty usually serves the purpose of passing a test or getting a better grade despite lacking the proper abilities or knowledge. In learning-related situations such as homework, where assignments are mandatory, cheating practices equally qualify as academic dishonesty. For perpetrators, these practices can be seen as shortcuts in which the willingness to invest the proper time and effort into learning is missing (Chow, 2021; Waltzer & Dahl, 2022). The interviews by Waltzer & Dahl (2022) reveal that students do perceive cheating as being wrong but this does not prevent them from engaging in at least one type of dishonest practice. While academic dishonesty is not a new phenomenon, it has been changing together with the development of new digital technologies (Anderman & Koenka, 2017; Ercegovac & Richardson, 2004). With the rapid growth in technologies, new forms of homework avoidance, such as copying and plagiarism, are developing (Evering & Moorman, 2012; Ma et al., 2008) summarized the findings of the 2006 U.S. surveys of the Josephson Institute of Ethics with the conclusion that the internet has led to a deterioration of ethics among students. In 2006, one-third of high school students had copied an internet document in the past 12 months, and 60% had cheated on a test. In 2012, these numbers were updated to 32% and 51%, respectively (Josephson Institute of Ethics, 2012). Further, 75% reported having copied another’s homework. Surprisingly, only a few studies have provided more recent evidence on the prevalence of academic dishonesty in middle and high schools. The results from colleges and universities are hardly comparable, and until now, this topic has not been addressed in international large-scale studies on schooling and school performance.

Despite the lack of representative studies, research has identified many factors in smaller and non-representative samples that might explain why some students engage in dishonest practices and others do not. These include male gender (Whitley et al., 1999), the “dark triad” of personality traits in contrast to conscientiousness and agreeableness (e.g., Cuadrado et al., 2021; Giluk & Postlethwaite, 2015), extrinsic motivation and performance/avoidance goals in contrast to intrinsic motivation and mastery goals (e.g., Anderman & Koenka, 2017; Krou et al., 2021), self-efficacy and achievement scores (e.g., Nora & Zhang, 2010; Yaniv et al., 2017), unethical attitudes, and low fear of being caught (e.g., Cheng et al., 2021; Kam et al., 2018), influenced by the moral norms of peers and the conditions of the educational context (e.g., Isakov & Tripathy, 2017; Kapoor & Kaufman, 2021). Similar factors have been reported regarding research on the causes of plagiarism (Husain et al., 2017; Moss et al., 2018). Further, the systematic review from Chiang et al. (2022) focused on factors of academic dishonesty in online learning environments. The analyses, based on the six-components behavior engineering, showed that the most prominent factors were environmental (effect of incentives) and individual (effect of motivation). Despite these intensive research efforts, there is still no overarching model that can comprehensively explain the interplay of these factors.

### Effects of homework engagement and digital dishonesty on school performance

In meta-analyses of schools, small but significant positive effects of homework have been found regarding learning and achievement (e.g., Baş et al., 2017; Chen & Chen, 2014; Fan et al., 2017). In their review, Fan et al. (2017) found lower effect sizes for studies focusing on the time or frequency of homework than for studies investigating homework completion, homework grades, or homework effort. In large surveys, such as PISA, homework measurement by estimating after-school working hours has been customary practice. However, this measure could hide some other variables, such as whether teachers even give homework, whether there are school or state policies regarding homework, where the homework is done, whether it is done alone, etc. (e.g., Fernández-Alonso et al., 2015, 2017). Trautwein (2007) and Trautwein et al. (2009) repeatedly showed that homework effort rather than the frequency or the time spent on homework can be considered a better predictor for academic achievement Effort and engagement can be seen as closely interrelated. Martin et al. (2017) defined engagement as the expressed behavior corresponding to students’ motivation. This has been more recently expanded by the notion of the quality of homework completion (Rosário et al., 2018; Xu et al., 2021). Therefore, it is a plausible assumption that academic dishonesty when doing homework is closely related to low homework effort and a low quality of homework completion, which in turn affects academic achievement. However, almost no studies exist on the effects of homework avoidance or academic dishonesty on academic achievement. Studies investigating the relationship between academic dishonesty and academic achievement typically use academic achievement as a predictor of academic dishonesty, not the other way around (e.g., Cuadrado et al., 2019; McCabe et al., 2001). The results of these studies show that low-performing students tend to engage in dishonest practices more often. However, high-performing students also seem to be prone to cheating in highly competitive situations (Yaniv et al., 2017).

### Present study and hypotheses

The present study serves three combined purposes.

First, based on the additional questionnaires integrated into the Program for International Student Assessment 2018 (PISA 2018) data collection in Switzerland, we provide descriptive figures on the frequency of homework effort and the various forms of digitally-supported homework avoidance practices.

Second, the data were used to identify possible factors that explain higher levels of digitally-supported homework avoidance practices. Based on our review of the literature presented in Section 1.1, we hypothesized (Hypothesis 1 – H1) that these factors include homework effort, age, gender, socio-economic status, and study program.

Finally, we tested whether digitally-supported homework avoidance practices were a significant predictor of test score performance. We expected (Hypothesis 2 – H2) that technology-related factors influencing test scores include not only those reported by Petko et al. (2017) but also self-reported engagement in digital dishonesty practices. .

## Methods

### Participants

Our analyses were based on data collected for PISA 2018 in Switzerland, made available in June 2021 (Erzinger et al., 2021). The target sample of PISA was 15-year-old students, with a two-phase sampling: schools and then students (Erzinger et al., 2019, p.7–8, OECD, 2019a). A total of 228 schools were selected for Switzerland, with an original sample of 5822 students. Based on the PISA 2018 technical report (OECD, 2019a), only participants with a minimum of three valid responses to each scale used in the statistical analyses were included (see Section 2.2). A final sample of 4771 responses (48% female) was used for statistical analyses. The mean age was 15 years and 9 months (*SD* = 3 months). As Switzerland is a multilingual country, 60% of the respondents completed the questionnaires in German, 23% in French, and 17% in Italian.

### Measures

#### Digital dishonesty in homework scale

This six-item digital dishonesty for homework scale assesses the use of digital technology for homework avoidance and copying (IC801 C01 to C06), is intended to work as a single overall scale for digital homework dishonesty practice constructed to include items corresponding to two types of dishonest practices from Pavela (1997), namely cheating and plagiarism (see Table 1). Three items target individual digital practices to avoid homework, which can be referred to as plagiarism (items 1, 2 and 5). Two focus more on social digital practices, for which students are cheating together with peers (items 4 and 6). One item target cheating as peer authorized plagiarism. Response options are based on questions on the productive use of digital technologies for homework in the common PISA survey (IC010), with an additional distinction for the lowest frequency option (6-point Likert scale). The scale was not tested prior to its integration into the PISA questionnaire, as it was newly developed for the purposes of this study.

#### Homework engagement scale

The scale, originally developed by Trautwein et al. (Trautwein, 2007; Trautwein et al., 2006), measures homework engagement (IC800 C01 to C06) and can be subdivided into two sub-scales: homework compliance and homework effort. The reliability of the scale was tested and established in different variants, both in Germany (Trautwein et al., 2006; Trautwein & Köller, 2003) and in Switzerland (Schnyder et al., 2008; Schynder Godel, 2015). In the adaptation used in the PISA 2018 survey, four items were positively poled (items 1, 2, 4, and 6), and two items were negatively poled (items 3 and 5) and presented with a 4-point Likert scale ranging from “Does not apply at all” to “Applies absolutely.” This adaptation showed acceptable reliability in previous studies in Switzerland (α = 0.73 and α = 0.78). The present study focused on homework effort, and thus only data from the corresponding sub-scale was analyzed (items 2 [I always try to do all of my homework], 4 [When it comes to homework, I do my best], and 6 [On the whole, I think I do my homework more conscientiously than my classmates]).

#### Demographics

Previous studies showed that demographic characteristics, such as age, gender, and socioeconomic status, could impact learning outcomes (Jacobs et al., 2002) and intention to use digital tools for learning (Tarhini et al., 2014). Gender is a dummy variable (ST004), with 1 for female and 2 for male. Socioeconomic status was analyzed based on the PISA 2018 index of economic, social, and cultural status (ESCS). It is computed from three other indices (OECD, 2019b, Annex A1): parents’ highest level of education (PARED), parents’ highest occupational status (HISEI), and home possessions (HOMEPOS). The final ESCS score is transformed so that 0 corresponds to an average OECD student. More details can be found in Annex A1 from PISA 2018 Results Volume 3 (OECD, 2019b).

#### Study program

Although large-scale studies on schools have accounted for the differences between schools, the study program can also be a factor that directly affects digital homework dishonesty practices. In Switzerland, 15-year-old students from the PISA sampling pool can be part of at least six main study programs, which greatly differ in terms of learning content. In this study, study programs distinguished both level and type of study: lower secondary education (gymnasial – *n* = 798, basic requirements – *n* = 897, advanced requirements – *n* = 1235), vocational education (classic – *n* = 571, with baccalaureate – *n* = 275), and university entrance preparation (*n* = 745). An “other” category was also included (*n* = 250). This 6-level ordinal variable was dummy coded based on the available CNTSCHID variable.

#### Technologies and schools

The PISA 2015 ICT (Information and Communication Technology) familiarity questionnaire included most of the technology-related variables tested by Petko et al. (2017): ENTUSE (frequency of computer use at home for entertainment purposes), HOMESCH (frequency of computer use for school-related purposes at home), and USESCH (frequency of computer use at school). However, the measure of student’s attitudes toward ICT in the 2015 survey was different from that of the 2012 dataset. Based on previous studies (Arpacı et al., 2021; Kunina-Habenicht & Goldhammer, 2020), we thus included INICT (Student’s ICT interest), COMPICT (Students’ perceived ICT competence), AUTICT (Students’ perceived autonomy related to ICT use), and SOIACICT (Students’ ICT as a topic in social interaction) instead of the variable ICTATTPOS of the 2012 survey.

#### Test scores

The PISA science, mathematics, and reading test scores were used as dependent variables to test our second hypothesis. Following Aparicio et al. (2021), the mean scores from plausible values were computed for each test score and used in the test score analysis.

### Data analyses

Our hypotheses aim to assess the factors explaining student digital homework dishonesty practices (H1) and test score performance (H2). At the student level, we used multilevel regression analyses to decompose the variance and estimate associations. As we used data for Switzerland, in which differences between school systems exist at the level of provinces (within and between), we also considered differences across schools (based on the variable CNTSCHID).

Data were downloaded from the main PISA repository, and additional data for Switzerland were available on forscenter.ch (Erzinger et al., 2021). Analyses were computed with Jamovi (v.1.8 for Microsoft Windows) statistics and R packages (GAMLj, lavaan).

## Results

### Additional scales for Switzerland

#### Digital dishonesty in homework practices

The digital homework dishonesty scale (6 items), computed with the six items IC801, was found to be of very good reliability overall (α = 0.91, ω = 0.91). After checking for reliability, a mean score was computed for the overall scale. The confirmatory factor analysis for the one-dimensional model reached an adequate fit, with three modifications using residual covariances between single items χ^2^(6) = 220, *p* < 0.001, TLI = 0.969, CFI = 0.988, RMSEA (Root Mean Square Error of Approximation) = 0.086, SRMR = 0.016).

**Table 1:** Frequencies of averaged digital dishonesty in homework (weighted data)

	Never	Almost never	Once or twice a month	Once or twice a week	Almost every day	Every day
… I partially copy things from the internet and modify them so that no one notices.	23.8%	29.0%	24.9%	15.0%	4.4%	2.9%
… I look on the internet for summaries or answers, so that I don’t have to do so much work myself.	20.3%	25.8%	27.9%	18.4%	5.0%	2.7%
… I copy friends’ answers, which they send me online or by phone.	15.7%	22.6%	28.1%	23.5%	6.9%	3.2%
… I do the homework on the internet together with others, even though I should be working on my own.	34.6%	22.9%	18.6%	15.4%	6.0%	2.6%
… I copy something from the internet and simply hand it in as my own work.	51.7%	19.7%	11.2%	10.3%	4.5%	2.7%
… I share my homework with others via the internet, so that people don’t have to do everything themselves.	32.4%	21.4%	19.7%	15.7%	6.6%	4.2%
Digital dishonesty (all practices considered)	7.6%	15.1%	27.7%	30.6%	12.1%	6.9%

On the one hand, the practice that was the least reported was copying something from the internet and presenting it as their own (51% never did). On the other hand, students were more likely to partially copy content from the internet and modify it to present as their own (47% did it at least once a month). Copying answers shared by friends was rather common, with 62% of the students reporting that they engaged in such practices at least once a month.

When all surveyed practices were taken together, 7.6% of the students reported that they had never engaged in digitally dishonest practices for homework, while 30.6% reported cheating once or twice a week, 12.1% almost every day, and 6.9% every day (Table 1).

#### Homework effort

The overall homework engagement scale consisted of six items (IC800), and it was found to be acceptably reliable (α = 0.76, ω = 0.79). Items 3 and 5 were reversed for this analysis. The homework compliance sub-scale had a low reliability (α = 0.58, ω = 0.64), whereas the homework effort sub-scale had an acceptable reliability (α = 0.78, ω = 0.79). Based on our rationale, the following statistical analyses used only the homework effort sub-scale. Furthermore, this focus is justified by the fact that the homework compliance scale might be statistically confounded with the digital dishonesty in homework scale.

Descriptive weighted statistics per item (Table 2) showed that while most students (80%) tried to complete all of their homework, only half of the students reported doing those diligently (53.3%). Most students also reported that they believed they put more effort into their homework than their peers (77.7%). The overall mean score of the composite scale was 2.81 (*SD* = 0.69).

**Table 2:** Frequencies of averaged homework engagement (weighted data)

	Does not apply at all	Does not apply to a great extent	Applies to a certain extent	Applies absolutely
I always try to do all of my homework.	5.0%	17.8%	44.8%	32.4%
When it comes to homework, I do my best.	5.6%	24.8%	51.2%	18.4%
On the whole, I think I do my homework more conscientiously than my classmates.	12.8%	35.0%	39.6%	12.7%

### Multilevel regression analysis: Predictors of digital dishonesty in homework (H1)

Mixed multilevel modeling was used to analyze predictors of digital homework avoidance while considering the effect of school (random component). Based on our first hypothesis, we compared several models by progressively including the following fixed effects: homework effort and personal traits (age, gender) (Model 2), then socio-economic status (Model 3), and finally, study program (Model 4). The results are presented in Table 3. Except for the digital homework dishonesty and homework efforts scales, all other scales were based upon the scores computed according to the PISA technical report (OECD, 2019a).

**Table 3:** Multilevel models explaining variations in students’ self-reported homework avoidance with digital resources

	Model 1	Model 2	Model 3	Model 4
	Fixed effects (β)			
Homework effort		-0.22^***^	-0.22^***^	-0.23^***^
Age		-0.03	-0.03	-0.08
Gender		0.24^***^	0.24^***^	0.23^***^
Socioeconomic status			-0.05	0.03
Study program				0.06^***^
	Models’ parameters			
Conditional R^2^	0.066	0.102	0.100	0.101
Marginal R^2^		0.034	0.036	0.044
b	2.56^***^	2.56^***^	2.56^***^	2.56^***^
SE b	0.025	0.025	0.025	0.025
95% CI	2.52, 2.61	2.51, 2.61	2.51, 2.61	2.51, 2.61
AIC	14465.49	13858.83	13715.70	13694.45
ICC	0.066	0.071	0.067	0.065

*Note*: * p < 0.05, ** p < 0.01, *** p < 0.001

We first compared variance components. Variance was decomposed into student and school levels. Model 1 provides estimates of the variance component without any covariates. The intraclass coefficient (ICC) indicated that about 6.6% of the total variance was associated with schools. The parameter *(b* = 2.56, *SE b* = 0.025*)* falls within the 95% confidence interval. Further, CI is above 0 and thus we can reject the null hypothesis. Comparing the empty model to models with covariates, we found that Models 2, 3 and 4 showed an increase in total explained variance to 10%. Variance explained by the covariates was about 3% in Models 2 and 3, and about 4% in Model 4. Interestingly, in our models, student socio-economic status, measured by the PISA index, never accounted for variance in digitally-supported dishonest practices to complete homework.

**Fig. 1:**
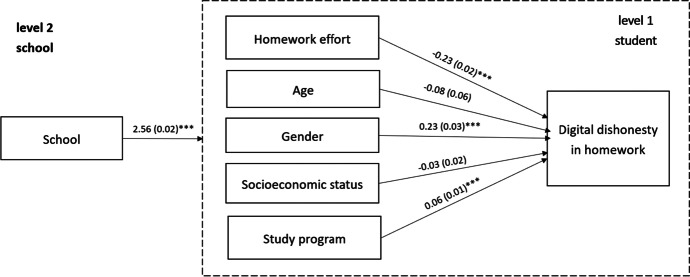
Summary of the two-steps Model 4 (estimates - β, with standard errors and significance levels, *** p < 0.001)

Further, model comparison based on AIC indicates that Model 4, including homework effort, personal traits, socio-economic status, and study program, was the better fit for the data. In Model 4 (Table 3; Fig. 1), we observed that homework effort and gender were negatively associated with digital dishonesty. Male students who invested less effort in their homework were more prone to engage in digital dishonesty. The study program was positively but weakly associated with digital dishonesty. Students in programs that target higher education were less likely to engage in digital dishonesty when completing homework.

### Multilevel regression analysis: Cheating and test scores (H2)

Our first hypothesis aimed to provide insights into characteristics of students reporting that they regularly use digital resources dishonestly when completing homework. Our second hypothesis focused on whether digitally-supported homework avoidance practices was linked to results of test scores. Mixed multilevel modeling was used to analyze predictors of test scores while considering the effect of school (random component). Based on the study by Petko et al. (2017), we compared several models by progressively including the following fixed effects ICT use (three measures) (Model 2), then attitude toward ICT (four measures) (Model 3), and finally, digital dishonesty in homework (single measure) (Model 4). The results are presented in Table 4 for science, Table 5 for mathematics, and Table 6 for reading.

**Table 4:** Multilevel models explaining variations in student test scores in science (standardized coefficients and model parameters)

	Model 1	Model 2	Model 3	Model 4
	Fixed effects (β)			
ENTUSE		1.84	-2.16	-1.02
HOMESCH		-12.05^***^	-10.80^***^	-9.87^***^
USESCH		-5.81^***^	-6.04^***^	-3.53^*^
INTICT			2.24	2.54^*^
COMPICT			6.35^***^	6.50^***^
AUTICT			9.95^***^	9.75^***^
SOIAICT			-7.68^***^	-5.93^***^
Digital dishonesty				-10.30^***^
	Models’ parameters			
Conditional R^2^	0.379	0.405	0.408	0.411
Marginal R^2^		0.025	0.051	0.069
b	495^***^	496.48^***^	497.68^***^	498^***^
SE b	3.82	3.79	3.64	3.55
95% CI	487, 502	489.05, 503.92	490.55, 504.81	491.05, 504.95
AIC	54619.43	52391.74	51309.22	51208.48
ICC	0.379	0.389	0.376	0.368

*Note*: * p < 0.05, ** p < 0.01, *** p < 0.001

Variance components were decomposed into student and school level. ICC for Model 1 indicated that 37.9% of the variance component without covariates was associated with schools.

Taking Model 1 as a reference, we observed an increase in total explained variance to 40.5% with factors related to ICT use (Model 2), to 40.8% with factors related to attitude toward ICT (Model 3), and to 41.1% with the single digital dishonesty factor. It is interesting to note that we obtained different results from those reported by Petko et al. (2017). In their study, they found significant effects on the explained variances of ENTUSE, USESCH, and ICTATTPOS but not of HOMESCH for Switzerland. In the present study (Model 3), HOMESCH and USESCH were significant predictors but not ENTUSE, and for attitude toward ICT, all but INTICT were significant predictors of the variance. However, factors corresponding to ICT use were negatively associated with test performance, as in the study by Petko et al. (2017). Similarly, all components of attitude toward ICT positively affected science test scores, except for students’ ICT as a topic in social interaction.

Based on the AIC values, Model 4, including ICT use, attitude toward ICT, and digital dishonesty, was the better fit for the data. The parameter (*b* = 498.00, *SE b* = 3.550) shows that our sample falls within the 95% confidence interval and that we can reject the null hypothesis. In this model, all factors except the use of ICT outside of school for leisure were significant predictors of explained variance in science test scores. These results are consistent with those reported by Petko et al. (2017), in which more frequent use of ICT negatively affected science test scores, with an overall positive effect of positive attitude toward ICT. Further, we observed that homework avoidance with digital resources strongly negatively affected performance, with lower performance associated with students reporting a higher frequency of engagement in digital dishonesty practices.

**Table 5:** Multilevel models explaining variations in student test scores in mathematics (standardized coefficients and model parameters)

	Model 1	Model 2	Model 3	Model 4
	Fixed effects (β)			
ENTUSE		1.82	-1.57	-0.56
HOMESCH		-10.45^***^	-9.88^***^	-9.05^***^
USESCH		-4.44^**^	-4.68^***^	-2.461
INTICT			0.380	0.648
COMPICT			5.440^***^	5.566^***^
AUTICT			7.157^***^	6.982^***^
SOIAICT			-3.416^**^	-1.876
Digital dishonesty				-9.102^***^
	Models’ parameters			
Conditional R^2^	0.388	0.408	0.410	0.412
Marginal R^2^		0.019	0.034	0.048
b	516^***^	516.84^***^	517.81^***^	518.09^***^
SE b	3.70	3.69	3.60	3.51
95% CI	508, 523	509.61, 524.07	510.76, 524.86	511.20,524.98
AIC	54139.46	52009.23	50985.87	50901.03
ICC	0.388	0.397	0.389	0.382

*Note*: * p < 0.05, ** p < 0.01, *** p < 0.001

For mathematics test scores, results from Models 2 and 3 showed a similar pattern than those for science, and Model 4 also explained the highest variance (41.2%). The results from Model 4 contrast with those found by Petko et al. (2017), as in this study, HOMESCH was the only significant variable of ICT use. Regarding attitudes toward ICT, only two measures (COMPICT and AUTICT) were significant positive factors in Model 4. As for science test scores, digital dishonesty practices were a significantly strong negative predictor. Students who reported cheating more frequently were more likely to perform poorly on mathematics tests.

**Table 6:** Multilevel models explaining variations in student test scores in reading (standardized coefficients and model parameters)

	Model 1	Model 2	Model 3	Model 4
	Fixed effects			
ENTUSE		-1.97	-5.07	-3.52^*^
HOMESCH		-13.12^***^	-11.23^***^	-9.97^***^
USESCH		-6.7^***^	-6.67^***^	-3.28^*^
INTICT			7.38^***^	7.79^***^
COMPICT			4.04^**^	4.23^*^
AUTICT			9.02^***^	8.75^***^
SOIAICT			-12.16^***^	-9.79^***^
Digital dishonesty				-13.94^***^
	Models’ parameters			
Conditional R^2^	0.381	0.410	0.413	0.422
Marginal R^2^		0.032	0.061	0.088
b	485	486.88	488.44	488.86
SE b	4.12	4.06	3.87	3.74
95% CI	477, 493	478.91, 494.84	480.86, 496.02	481.54, 496.18
AIC	55305.13	53003.48	51871.13	51705.75
ICC	0.381	0.390	0.375	0.366

*Note*: * p < 0.05, ** p < 0.01, *** p < 0.001

The analyses of PISA test scores for reading in Model 2 was similar to that of science and mathematics, with ENTUSE being a non-significant predictor when we included only measures of ICT use as predictors. In Model 3, contrary to the science and mathematics test scores models, in which INICT was non-significant, all measures of attitude toward ICT were positively significant predictors. Nevertheless, as for science and mathematics, Model 4, which included digital dishonesty, explained the greater variance in reading test scores (42.2%). We observed that for reading, all predictors were significant in Model 4, with an overall negative effect of ICT use, a positive effect of attitude toward ICT—except for SOIAICT, and a negative effect of digital dishonesty on test scores. Interestingly, the detrimental effect of using digital resources to engage in dishonest homework completion was the strongest in reading test scores.

## Discussion

In this study, we were able to provide descriptive statistics on the prevalence of digital dishonesty among secondary students in the Swiss sample of PISA 2018. Students from this country were selected because they received additional questions targeting both homework effort and the frequency with which they engaged in digital dishonesty when doing homework. Descriptive statistics indicated that fairly high numbers of students engage in dishonest homework practices, with 49.6% reporting digital dishonesty at least once or twice a week. The most frequently reported practice was copying answers from friends, which was undertaken at least once a month by more than two-thirds of respondents. Interestingly, the most infamous form of digital dishonesty, that is plagiarism by copy-pasting something from the internet (Evering & Moorman, 2012), was admitted to by close to half of the students (49%). These results for homework avoidance are close to those obtained by previous research on digital academic plagiarism (e.g., McCabe et al., 2001).

We then investigated what makes a cheater, based on students’ demographics and effort put in doing their homework (H1), before looking at digital dishonesty as an additional ICT predictor of PISA test scores (mathematics, reading, and science) (H2).

The goal of our first research hypothesis was to determine student-related factors that may predict digital homework avoidance practices. Here, we focused on factors linked to students’ personal characteristics and study programs. Our multilevel model explained about 10% of the variance overall. Our analysis of which students are more likely to digital resources to avoid homework revealed an increased probability for male students who did not put much effort into doing their homework and who were studying in a program that was not oriented toward higher education. Thus, our findings tend to support results from previous research that stresses the importance of gender and motivational factors for academic dishonesty (e.g., Anderman & Koenka, 2017; Krou et al., 2021). Yet, as our model only explained little variance and more research is needed to provide an accurate representation of the factors that lead to digital dishonesty. Future research could include more aspects that are linked to learning, such as peer-related or teaching-related factors. Possibly, how closely homework is embedded in the teaching and learning culture may play a key role in digital dishonesty. Additional factors might be linked to the overall availability and use of digital tools. For example, the report combining factors from the PISA 2018 school and student questionnaires showed that the higher the computer–student ratio, the lower students scored in the general tests (OECD, 2020b). A positive association with reading disappeared when socio-economic background was considered. This is even more interesting when considering previous research indicating that while internet access is not a source of divide among youths, the quality of use is still different based on gender or socioeconomic status (Livingstone & Helsper, 2007). Thus, investigating the usage-related “digital divide” as a potential source of digital dishonesty is an interesting avenue for future research (Dolan, 2016).

Our second hypothesis considered that digital dishonesty in homework completion can be regarded as an additional ICT-related trait and thus could be included in models targeting the influence of traditional ICT on PISA test scores, such as Petko et al. (2017) study. Overall, our results on the influence of ICT use and attitudes toward ICT on test scores are in line with those reported by Petko et al. (2017). Digital dishonesty was found to negatively influence test scores, with a higher frequency of cheating leading to lower performance in all major PISA test domains, and particularly so for reading. For each subject, the combined models explained about 40% of the total variance.

### Conclusions and recommendations

Our results have several practical implications. First, the amount of cheating on homework observed calls for new strategies for raising homework engagement, as this was found to be a clear predictor of digital dishonesty. This can be achieved by better explaining the goals and benefits of homework, the adverse effects of cheating on homework, and by providing adequate feedback on homework that was done properly. Second, teachers might consider new forms of homework that are less prone to cheating, such as doing homework in non-digital formats that are less easy to copy digitally or in proctored digital formats that allow for the monitoring of the process of homework completion, or by using plagiarism software to check homework. Sometimes, it might even be possible to give homework and explicitly encourage strategies that might be considered cheating, for example, by working together or using internet sources. As collaboration is one of the 21st century skills that students are expected to develop (Bray et al., 2020), this can be used to turn cheating into positive practice. There is already research showing the beneficial impact of computer-supported collaborative learning (e.g., Janssen et al., 2012). Zhang et al. (2011) compared three homework assignment (creation of a homepage) conditions: individually, in groups with specific instructions, and in groups with general instructions. Their results showed that computer supported collaborative homework led to better performance than individual settings, only when the instructions were general. Thus, promoting digital collaborative homework could support the development of students’ digital and collaborative skills.

Further, digital dishonesty in homework needs to be considered different from cheating in assessments. In research on assessment-related dishonesty, cheating is perceived as a reprehensible practice because grades obtained are a misrepresentation of student knowledge, and cheating “implies that efficient cheaters are good students, since they get good grades” (Bouville, 2010, p. 69). However, regarding homework, this view is too restrictive. Indeed, not all homework is graded, and we cannot know for sure whether students answered this questionnaire while considering homework as a whole or only graded homework (assessments). Our study did not include questions about whether students displayed the same attitudes and practices toward assessments (graded) and practice exercises (non-graded), nor did it include questions on how assessments and homework were related. By cheating on ungraded practice exercises, students will primarily hamper their own learning process. Future research could investigate in more depth the kinds of homework students cheat on and why.

Finally, the question of how to foster engaging homework with digital tools becomes even more important in pandemic situations. Numerous studies following the switch to home schooling at the beginning of the 2020 COVID-19 pandemic have investigated the difficulties for parents in supporting their children (Bol, 2020; Parczewska, 2021); however, the question of digital homework has not been specifically addressed. It is unknown whether the increase in digital schooling paired with discrepancies in access to digital tools has led to an increase in digital dishonesty practices. Data from the PISA 2018 student questionnaires (OECD, 2020a) indicated that about 90% of students have a computer for schoolwork (OECD average), but the availability per student remains unknown. Digital homework can be perceived as yet another factor of social differences (see for example Auxier & Anderson, 2020; Thorn & Vincent-Lancrin, 2022).

### Limitations and directions

The limitations of the study include the format of the data collected, with the accuracy of self-reports to mirror actual practices restricted, as these measures are particularly likely to trigger response bias, such as social desirability. More objective data on digital dishonesty in homework-related purposes could, for example, be obtained by analyzing students’ homework with plagiarism software. Further, additional measures that provide a more complete landscape of contributing factors are necessary. For example, in considering digital homework as an alternative to traditional homework, parents’ involvement in homework and their attitudes toward ICT are factors that have not been considered in this study (Amzalag, 2021). Although our results are in line with studies on academic digital dishonesty, their scope is limited to the Swiss context. Moreover, our analyses focused on secondary students. Results might be different with a sample of younger students. As an example, Kiss and Teller (2022) measured primary students cheating practices and found that individual characteristics were not a stable predictor of cheating between age groups. Further, our models included school as a random component, yet other group variables, such as class and peer groups, may well affect digital homework avoidance strategies.

The findings of this study suggest that academic dishonesty when doing homework needs to be addressed in schools. One way, as suggested by Chow et al. (2021) and Djokovic et al. (2022), is to build on students’ practices to explain which need to be considered cheating. This recommendation for institutions to take preventive actions and explicit to students the punishment faced in case of digital academic behavior was also raised by Chiang et al. (2022). Another is that teachers may consider developing homework formats that discourage cheating and shortcuts (e.g., creating multimedia documents instead of text-based documents, using platforms where answers cannot be copied and pasted, or using advanced forms of online proctoring). It may also be possible to change homework formats toward more open formats, where today’s cheating practices are allowed when they are made transparent (open-book homework, collaborative homework). Further, experiences from the COVID-19 pandemic have stressed the importance of understanding the factors related to the successful integration of digital homework and the need to minimize the digital “homework gap” (Auxier & Anderson, 2020; Donnelly & Patrinos, 2021). Given that homework engagement is a core predictor of academic dishonesty, students should receive meaningful homework in preparation for upcoming lessons or for practicing what was learned in past lessons. Raising student’s awareness of the meaning and significance of homework might be an important piece of the puzzle to honesty in learning.

## Data Availability

The data that support the findings of this study are openly available in SISS base at 10.23662/FORS-DS-1285-1, reference number 1285.

## Appendix 1

List of abbreviations related to PISA datasets

AUTICT: students’ perceived autonomy related to ICT use
COMPICT: students’ perceived ICT competence
ENTUSE: frequency of computer use at home for entertainment purposes
ESCS: index of economic, social, and cultural status (computed from PARED, HISEI and HOMEPOS)
HISEI: parents’ highest occupational status
HOMEPOS: home possessions
HOMESCH: frequency of computer use for school-related purposes at home
IC800: digital cheating for homework items for Switzerland
IC801: homework engagement items for Switzerland
ICTATTPOS: positive attitude towards ICT as a learning tool
INICT: student’s ICT interest
PARED: parents’ highest level of education
SOIACICT: students’ ICT as a topic in social interaction
USESCH: frequency of computer use at school
